# Three-territory sign in infective endocarditis and concomitant stroke: a retrospective analysis on characteristics and associated factors

**DOI:** 10.1186/s42466-026-00493-5

**Published:** 2026-04-20

**Authors:** Hannah Schuermann, Regina von Rennenberg, Christoph Riegler, Simon Hellwig, Helena Stengl, Simon Litmeier, Jan F. Scheitz, Wolfram Doehner, Heinrich Audebert, Tim Bastian Braemswig, Christian H. Nolte

**Affiliations:** 1 Klinik und Hochschulambulanz für Neurologie mit experimenteller Neurologie, Charité – Universitätsmedizin Berlin, corporate member of Freie Universität Berlin and Humboldt- Universität zu Berlin, Hindenburgdamm 30, Berlin, 12200 Germany; 2 Center for Stroke Research Berlin, Charité-Universitätsmedizin Berlin, Berlin, Germany; 3 Berlin Institute of Health at Charité – Universitätsmedizin Berlin, Charitéplatz 1, Berlin, 10117 Germany; 4 Department Cardiology (Virchow Klinikum), Deutsches Herzzentrum der Charité, Charité - Universitätsmedizin Berlin, Berlin, Germany; 5 Berlin Institute of Health Center for Regenerative Therapies, Charité - Universitätsmedizin Berlin, Charitéplatz 1, Berlin, 10117 Germany; 6 German Centre for Cardiovascular Research (DZHK)Partner-Site Berlin, Potsdamer Str. 58, Berlin, 10785 Germany; 7 Department of Neurology with experimental Neurology, Charité – Universitätsmedizin Berlin, corporate member of Freie Universität Berlin, Humboldt Universität zu Berlin, Hindenburgdamm 30, Berlin, 12203 Germany

**Keywords:** Infective endocarditis, Stroke pattern, Cerebral imaging, Three-territory sign

## Abstract

**Background:**

Infective endocarditis (IE) is a rare yet challenging cause of acute stroke. A stroke imaging pattern with ischemic lesions in all three major cerebral vascular supply territories (three-territory sign), is considered typical for IE. However, data on its frequency and significance are scarce.

**Methods:**

Data on IE patients with concomitant acute stroke admitted to three tertiary care hospitals in Berlin, Germany, between 2017 and 2023 were retrospectively analyzed. Presence of the three-territory sign was evaluated on cerebral magnetic resonance imaging (cMRI). Presence and distribution of accompanying hemorrhagic stroke, cerebral microbleeds (CMB) and chronic infarcts were evaluated, too. Bivariate and multivariable logistic regression analyses were performed to identify variables associated with three-territory sign.

**Results:**

We identified 135 patients with IE and acute stroke on cMRI (median age 67 years [56–76], 31.9% female). Three-territory sign was present in 86/135 patients (63.7%). Three-territory sign was independently associated with detection of Staphylococcus aureus [adjusted odds ratio (aOR) = 3.98 (95% CI 1.35–11.75)] and additional extracerebral arterial embolism [aOR = 4.78 (95% CI 1.77–12.91)] but not with presence or distribution of hemorrhagic stroke, CMB or chronic infarcts. In-hospital mortality was higher in IE patients with three-territory sign compared to those without (31.4% vs. 12.2%; *p* = 0.01).

**Conclusions:**

Three-territory sign is common but not obligatory in IE patients with acute ischemic stroke. cMRI will rather depict this stroke pattern in the more severely diseased IE patients as three-territory sign makes detection of a particularly virulent pathogen (Staphylococcus aureus) more likely and indicates higher severity of disease.

**Trial registration:**

Not applicable.

## Introduction

Infective endocarditis (IE) is a rare cause of acute stroke [1]. However, acute stroke (ischemic and/or hemorrhagic) due to IE is associated with increased morbidity and mortality compared to IE patients without stroke [2–4]. Current literature provides some insights on factors associated with stroke in IE (i.e. pathogens, affected heart valves, complications) [5–8], but information on the characteristics and significance of the infarct pattern on cerebral magnetic resonance imaging (cMRI) is very limited [9]. Characteristics of the infarct pattern could provide valuable support in clinical decision making, i.e. prompting to think of IE, suspecting a specific pathogen, and assessing the further course of the disease.

A characteristic infarct pattern is the previously described ‘three-territory sign’ (an infarct pattern with lesions in all three vascular supply territories, including territory of the left and right internal carotid artery, and the vertebrobasilar territory). So far, the three-territory sign has been investigated in the setting of stroke due to malignancy-associated coagulopathy [10, 11]. However, the three-territory sign is not exclusive to malignancy but may also indicate other sources of proximal emboli, in particular underlying IE [6, 12].

So far, little is known about the frequency of the three-territory sign in IE patients with concomitant stroke and its association with relevant characteristics of the underlying disease (i.e. causative pathogens, heart valves involved, prognosis).

We therefore aimed to assess the frequency, characteristics and factors associated with the three-territory sign in patients with IE and acute stroke.

## Methods

This retrospective cohort study analyzed consecutive patients with IE and concomitant acute (ischemic and/or hemorrhagic) stroke undergoing cerebral magnetic resonance imaging (cMRI) between January 2017 and December 2023 at three academic tertiary care hospitals of the Charité – Universitätsmedizin Berlin, Germany.

### Patients

**Fig. 1:**
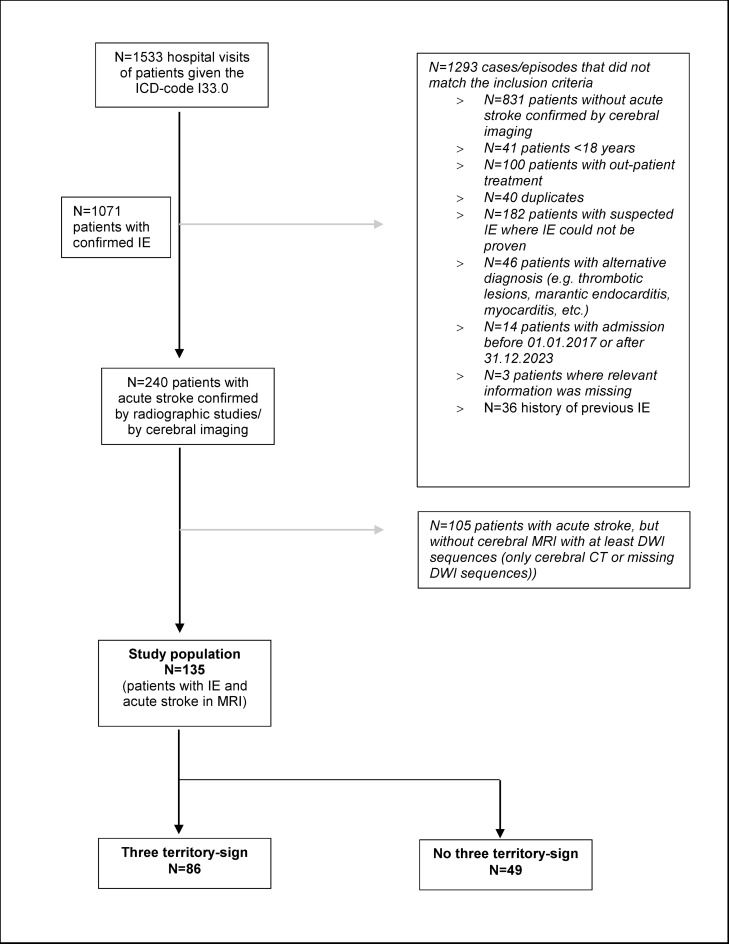
Flowchart. IE indicates infective endocarditis, MRI magnetic resonance imaging, DWI Diffusion weighted imaging, CT computed Tomography

We identified patients with IE using the ICD-10 code for IE (ICD-10 I33.0) as previously described [7]. Inclusion criteria were: (1) age ≥18 years, (2) inpatient treatment, (3) acute episode of IE, and (4) concomitant radiological evidence of acute ischemic or hemorrhagic lesions on cMRI regardless of clinical manifestations. MRI had to include diffusion weighted imaging (DWI) to assess acute ischemic lesions. We reviewed all cases regarding the modified Duke criteria according to 2023 European Society of Cardiology (ESC) Guidelines for the management of endocarditis [13]. Patients who did not fulfill these criteria were excluded as depicted in Fig. 1 (flow chart).

### Clinical data

Data were retrieved from hospital records and included physician’s letters, laboratory, microbiologic and echocardiographic findings (transesophageal echocardiography, transthoracic echocardiography), carotid duplex ultrasound, coronary angiography and radiological results (cMRI, cerebral CT, magnetic resonance angiography (MRA), computed tomography angiography (CTA)).

### Cerebral MRI

cMRI scans were assessed (1) in clinical routine and (2) by an additional rater of this study (HSc). In cases of disagreement, a third rater (TBB or CHN) was consulted. The first available cMRI was used for analysis.

Acute ischemic brain lesions were assessed on diffusion weighted imaging (DWI). Three vascular supply territories were differentiated according to the literature: Territory of the left internal carotid artery, territory of the right internal carotid artery, and the vertebrobasilar territory [14, 15].

Intracerebral hemorrhage (ICH), cerebral microbleeds (CMBs), subarachnoid hemorrhage (SAH), cortical superficial siderosis (CSS) as well as spontaneous hemorrhagic transformation (HT) of ischemic stroke were assessed on hemorrhage-sensitive MRI sequences (gradient echo (GRE) or susceptibility weighted imaging (SWI)) [16, 17]. HT secondary to reperfusion therapy (i.e. intravenous thrombolysis, IVT, or endovascular treatment, EVT) was assessed as a separate entity. Distribution of CMBs was categorized according to established patterns (infratentorial, deep, lobar, or mixed) [18]. Chronic ischemic stroke lesions and white matter hyperintensities were assessed on fluid-attenuated inversion recovery images (FLAIR) using the Fazekas score [19]. Chronic ischemic lesions were classified into (1) single, (2) scattered (several lesions in one vascular territory), or (3) multiple (several lesions in more than one vascular territory) as previously reported [14]. The type of chronic infarct was distinguished into (1) lacunar (following the Standards for Reporting Vascular Changes on Neuroimaging (STRIVE)) [17], (2) embolic (territorial or partial territorial infarction), (3) both, or (4) other/no differentiation possible.

### Definitions

IE (possible/definite) and pathogens were categorized according to the current version of the ESC Guidelines for the management of endocarditis [13]. Pathogens not listed as ‘major criterion 1’ were classified as atypical [13].

Stroke was considered symptomatic in IE patients with acute DWI lesions and focal neurological deficits. In patients with IE and acute DWI lesions but without focal neurological deficits, stroke was considered asymptomatic. Patients with altered mental status, and patients who were intubated and/or sedated were classified as not assessable.

Symptomatic carotid artery stenosis was defined as stenosis > 50% ipsilateral to at least one ischemic lesion on cMRI [20].

The following were considered as intracardiac foreign material: prosthetic heart valve, implantable electronic devices (e.g. pacemaker, implantable cardioverter defibrillator) and any further foreign material inside the heart (e.g. MitraClip). Intracardiac foreign material was classified as affected by IE if vegetations were found on the material in heart imaging.

### Statistics

Chi-squared test and Fisher’s exact test were used for comparison of nominal variables, Mann-Whitney U test was used for comparison of continuous variables. Unadjusted logistic regression analysis for three-territory sign was conducted with all variables available. In a second step, we performed multivariable logistic regression analysis adjusting for variables that showed statistically significant associations in bivariate analysis. Age and sex were added to the model. Multicollinearity was assessed for variables included in the adjusted logistic regression model (besides age and sex) by calculation of the variance inflation factor (VIF). In addition, we performed a multivariable logistic regression analysis for in-hospital mortality with adjustment for age and sex.

Statistical analysis was conducted using SPSS version 27.0 (SPSS Inc., Chicago, IL).

### Ethics

Approval for the study was granted by the Ethics committee of the Charité – Universitätsmedizin Berlin (No. EA2/289/23) on Jan 17th 2024.

The study follows the Strengthening the Reporting of Observational Studies in Epidemiology (STROBE) recommendations for observational studies [21].

## Results

In total, 240 IE patients were diagnosed with acute stroke (ischemic and/or hemorrhagic) as confirmed by cerebral CT/MRI. Out of these 240 patients, 135 (56.3%) underwent cerebral MRI including a DWI sequence. They constitute the final study population (see Flowchart, Fig. 1). All 135 patients had an ischemic stroke and 16 patients also had accompanying hemorrhagic stroke (ICH and/or SAH). Sex, median age and in-hospital mortality did not differ significantly between IE patients that underwent cMRI and patients without cMRI.

Median age was 67 years (IQR: 56–76) and 43/135 (31.9%) patients were female (see Table 1). Intravenous drug abuse was present in 6 patients (4.5%). Median length of stay was 24 days (IQR: 13–36).

**Table 1:** Bivariate analysis for the presence of three-territory sign – Baseline characteristics

Parameter	All patients(*N*=135)	*N*=135		*p-value*	Odds Ratio &95% CI
		Three-territory sign (N=86)	No three territory sign (N=49)		
**Demographics**					
Age, years, (median, IQR)	67 (56–76)	68 (55–76)	67 (57–75)	0.70	1.00 (0.98–1.03.98.03)
Sex, male (n, %)	92 (68.1%)	56 (65.1%)	36 (73.5%)	0.32	1.48 (0.68–3.22.68.22)
**Risk factors and Comorbidities**					
Cardiovascular risk factors (*N*=134) Arterial hypertension Dyslipidemia Diabetes mellitus Coronary artery disease Atrial fibrillation	91 (67.9%) 62 (46.3%) 36 (26.9%) 51 (37.8%) 43 (32.1%)	57 (66.3%) 37 (43.0%) 25 (29.1%) 32 (37.2%) 30 (34.9%)	34 (70.8%) 25 (52.1%) 11 (22.9%) 19 (38.8%) 13 (27.1%)	0.59 0.31 0.44 0.86 0.35	0.81 (0.38–1.74.38.74) 0.70 (0.34–1.41.34.41) 1.38 (0.61–3.13.61.13) 0.94 (0.45–1.93.45.93) 1.44 (0.66–3.13.66.13)
Congenital heart disease (*N*=134)	9 (6.7%)	4 (4.7%)	5 (10.4%)	0.20	0.42 (0.12–1.64.12.64)
Intracardiac foreign material (*N*=134)	36 (26.9%)	23 (26.7%)	13 (27.1%)	0.97	0.98 (0.44–2.18.44.18)
Stroke in medical history (*N*=134)	11 (8.2%)	7 (8.1%)	4 (8.3%)	0.97	0.98 (0.27–3.52.27.52)
IE in medical history (*N*=134)	5 (3.7%)	3 (3.5%)	2 (4.2%)	0.84	0.83 (0.13–5.16.13.16)
Intravenous drug abuse (*N*=134)	6 (4.5%)	4 (4.7%)	2 (4.2%)	0.90	1.12 (0.20–6.36.20.36)
Malignancy (*N*=134)	23 (17.2%)	17 (19.8%)	6 (12.5%)	0.29	1.73 (0.63–4.72.63.72)
**Outcome**					
In-hospital death	33 (24.4%)	27 (31.4%)	6 (12.2%)	**0.01**	**3.28 (1.25–8.64.25.64)**

IE indicates infective endocarditis

Considering baseline characteristics patients with and without three-territory sign significantly differed regarding mortality. Demographics and risk factors/comorbidities did not differ significantly between patients with and without three-territory sign

### Parameters related to IE

According to the modified Duke criteria[13], 89/135 (66.7%) had definite IE and 46/135 (33.3%) had possible IE. Blood cultures were positive in 86/135 (63.7%) patients. A typical lesion suggestive of IE on cardiac imaging was identified in 128 patients (94.8%). The mitral valve was the most commonly affected valve (56.3%), followed by the aortic valve (51.1%). The most frequently identified pathogen was Staphylococcus aureus (35.6%). In 104/135 patients extracerebral diagnostic imaging studies (i.e. CT or ultrasound of thorax and abdomen) were performed and about half of these patients had extracerebral arterial embolic events (55/104; 52.9%).

### Parameters related to stroke

Stroke was the admission diagnosis in 40/135 patients (29.6%) and IE in 24/135 (17.8%). All other patients (72/135; 52.9%) were admitted with unspecific symptoms/other diagnoses. Focal neurological deficits were present in 68.8% and absent in 6.2% of patients. Focal neurological deficits could not be ruled out definitively on the basis of documentation available in 25.0%. Reperfusion therapy was applied in nine patients: Four patients received IVT, three patients underwent EVT, and two patients had both IVT and EVT combined (bridging). Follow-up imaging revealed secondary intracerebral bleeding in 3/9 patients (IVT or EVT).

### MRI findings, frequency of three-territory sign

**Fig. 2:**
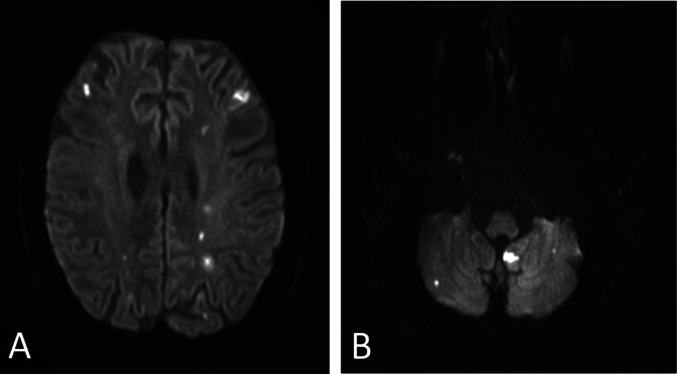
Three-territory sign on cMRI. Acute ischemic stroke lesions on DWI sequence of a patient with infective endocarditis with acute ischemic lesions in the territory of the left and right carotid artery (A) and the vertebrobasilar territory (B).

Three-territory sign was detected in 86/135 patients (63.7%) (Fig. 2). The three individual vascular territories were affected with similar frequency (ICA left 81.5%; ICA right 83.7%; posterior 83.7%).

Blood-sensitive sequences (GRE or SWI) were available in 108/135 (80.0%) patients. Secondary hemorrhagic transformation of primarily ischemic stroke occurred in 38/108 (35.2%) patients. Primary ICH occurred in 4/108 (3.7%) patients; all four patients had a lobar ICH location. SAH occurred in 13/108 patients (12.0%). CMBs were detected in 39/108 patients (36.1%). CMB distribution was predominantly lobar (36/39 patients, 92.3%).

Chronic ischemic infarcts were present in 32.1% of patients (data was available in 131/135 patients).

### Clinical outcome

In hospital mortality was 24.4% (33/135), 76/135 patients (56.3%) were transferred to rehabilitation, nursing home or another hospital for further therapy, and 26/135 (19,3%) were discharged home.

### Factors associated with the three-territory sign

Tables 1 and 2 show baseline characteristics, IE- and stroke-specific variables, imaging findings and functional outcome for patients with and without three-territory sign.

**Table 2:** Bivariate analysis for the presence of three-territory sign – Disease related factors

Parameter	All patients (*N* = 135)	*N* = 135		*p*-value	Odds Ratio & 95% CI
		Three-territory sign *(N = 86)*	No three territory sign *(N = 49)*		
**Parameters related to IE**					
Pathogen isolated in blood culture Staphylococcus aureus Oral Streptococci Enterococcus faecalis Streptococcus gallolyticus HACEK Atypical No pathogen identified	48 (35.6%) 11 (8.1%) 14 (10.4%) 5 (3.7%) 1 (0.7%) 23 (17.0%) 33 (24.4%)	39 (45.3%) 6 (7.0%) 3 (3.5%) 2 (2.3%) 0 (0%) 16 (18.6%) 20 (23.3%)	9 (18.4%) 5 (10.2%) 11 (22.4%) 3 (6.1%) 1 (2.0%) 7 (14.3%) 13 (26.5%)	**0.002**	**2.82 (1.03–7.71)** 0.78 (0.20–3.09) **0.18 (0.04–0.76)** 0.43 (0.06–2.96) n.a. 1.49 (0.48–4.60) Reference
Valves affected Mitral valve Aortic valve Tricuspid valve Pulmonary valve Multiple valves affected Intracardiac foreign material affected (*N* = 36)	76 (56.3%) 69 (51.1%) 2 (1.5%) 0 (0%) 12 (8.9%) 28 (77.8%)	54 (62.8%) 42 (48.8%) 1 (1.2%) 0 (0%) 11 (12.8%) 17 (73.9%)	22 (44.9%) 27 (55.1%) 1 (2.0%) 0 (0%) 1 (2.0%) 11 (84.6%)	**0.04** 0.48 0.69 n.a. **0.04** 0.46	**2.07 (1.02–4.22)** 0.78 (0.39–1.57) 0.57 (0.04–9.23) n.a. 7.04 (0.88–56.29) 0.52 (0.09–3.03)
In-hospital surgical valve repair	76 (56.3%)	51 (59.3%)	25 (51.0%)	0.35	1.40 (0.69–2.84)
Pathogen valve (*N* = 72) Staphylococcus aureus Oral Streptococci Enterococcus faecalis Streptococcus gallolyticus HACEK Atypical No pathogen found/No differentiation possible	9 (12.5%) 2 (2.8%) 4 (5.6%) 0 (0%) 0 (0%) 13 (18.1%) 44 (61.1%)	8 (16.0%) 1 (2.0%) 0 (0%) 0 (0%) 0 (0%) 8 (16.0%) 33 (66.0%)	1 (4.5%) 1 (4.5%) 4 (18.2%) 0 (0%) 0 (0%) 5 (22.7%) 11 (50.0%)	**0.02**	2.67 (0.30–23.78.30.78) 0.33 (0.02–5.79) n.a. n.a. n.a. 0.53 (0.14–1.98) Reference
Focus/Entry site of infection (suspected) (*N* = 126) Dental/Oral Spine Catheter associated Skin associated Other Multiple foci/entry sites No focus/entry site identified	16 (12.7%) 8 (6.3%) 14 (11.1%) 13 (10.3%) 14 (11.1%) 13 (10.3%) 48 (38.1%)	12 (14.5%) 5 (6.0%) 11 (13.3%) 10 (12.0%) 8 (9.6%) 7 (8.4%) 30 (36.1%)	4 (9.3%) 3 (7.0%) 3 (7.0%) 3 (7.0%) 6 (14.0%) 6 (14.0%) 18 (41.9%)	0.69	1.80 (0.50–6.43) 1.00 (0.21–4.69) 2.20 (0.54–8.96) 2.00 (0.49–8.24) 0.80 (0.24–2.68) 0.70 (0.20–2.41) Reference
Extracerebral arterial embolic events (*N* = 104) Kidney Spleen Lung Liver Extremities Other	55 (52.9%) 20 (19.2%) 43 (41.3%) 9 (8.7%) 4 (3.8%) 11 (10.6%) 2 (1.9%)	43 (66.2%) 15 (23.1%) 36 (55.4%) 7 (10.8%) 4 (6.2%) 9 (13.8%) 1 (1.5%)	12 (30.8%) 5 (12.8%) 7 (17.9%) 2 (5.1%) 0 (0%) 2 (5.1%) 1 (2.6%)	**< 0.001 ** 0.199 **< 0.001** 0.32 0.11 0.16 0.71	**4.398 (1.875–10.312) ** 2.040 (0.678–6.140) **5.68 (2.19–14.72)** 2.23 (0.44–11.34) n.a. 2.97 (0.61–14.54) 0.59 (0.04–9.77)
**Parameters related to stroke**					
Imaging proof of (may present simultaneously) Ischemic stroke Hemorrhagic stroke (ICH, SAH) (*N* = 108)	135 (100%) 16 (14.8%)	86 (100%) 13 (19.1%)	49 (100%) 3 (7.5%)	n.a. 0.10	n.a. 2.92 (0.78–10.94)
Symptomatic stroke (*N* = 112) Symptomatic stroke Asymptomatic (assessed) Not assessable	77 (68.8%) 7 (6.3%) 28 (25.0%)	53 (70.7%) 2 (2.7%) 20 (26.7%)	24 (64.9%) 5 (13.5%) 8 (21.6%)	0.08	5.52 (01.00–30.50.00.50) Reference 6.25 (1.00–39.09.00.09)
**Laboratory Data** ^**‡**^					
CRP on admission, mg/l (median, IQR) (*N* = 123)	104.20 (41.10–189.90.10.90)	122.50 (45.60–232.60.60.60)	66.85 (35.28–147.63.28.63)	**0.02**	**1.01 (1.00–1.01.00.01)**
Leucocyte count on admission,/nl (median, IQR) (*N* = 124)	10.84 (8.35–15.35)	10.70 (8.22–15.11)	12.03 (8.37–16.00.37.00)	0.42	0.95 (0.90–1.01)
**MRI findings**					
Secondary hemorrhagic transformation (*N* = 108)	38 (35.2%)	28 (41.2%)	10 (25.0%)	0.09	2.10 (0.89–4.98)
Cerebral microbleeds (CMBs) (*N* = 108) Distribution of CMBs (*N* = 39) Infratentorial Lobar Deep Strictly lobar Strictly deep or mixed	39 (36.1%) 13 (33.3%) 36 (92.3%) 19 (48.7%) 16 (41.0%) 22 (56.4%)	25 (36.8%) 8 (32.0%) 24 (96.0%) 13 (52.0%) 10 (40.0%) 15 (60.0%)	14 (35.0%) 5 (35.7%) 12 (85.7%) 6 (42.9%) 6 (42.9%) 7 (50.0%)	0.85 0.81 0.25 0.58 0.86 0.55	1.08 (0.48–2.44) 0.85 (0.21–3.36) 4.00 (0.33–48.65) 1.44 (0.39–5.39) 0.89 (0.24–3.35) 1.50 (0.40–5.61)
Number CMBs (median, IQR)	0 (0–2)	0 (0–2)	0 (0–1)	0.77	0.99 (0.97–1.01)
Subarachnoid hemorrhage (*N* = 108)	13 (12.0%)	10 (14.7%)	3 (7.5%)	0.27	2.13 (0.55–8.24)
Intracerebral hemorrhage (*N* = 108)	4 (3.7%)	4 (5.9%)	0 (0%)	0.12	n.a.
Chronic ischemic infarcts (*N* = 131)	42 (32.1%)	26 (31.7%)	16 (32.7%)	0.91	0.96 (0.45–2.04)
Pattern chronic infarcts (*N* = 42) Single Scattered Multiple	17 (40.5%) 9 (21.4%) 16 (38.1%)	12 (46.2%) 3 (11.5%) 11 (42.3%)	5 (31.3%) 6 (37.5%) 5 (31.3%)	0.14	Reference 0.21 (0.037–1.18) 0.92 (0.21–4.05)
Type chronic infarcts (*N* = 42) Lacune Embolic Lacune and Embolic Other	16 (38.1%) 11 (26.2%) 2 (4.8%) 13 (31.0%)	12 (46.2%) 7 (26.9%) 1 (3.8%) 6 (23.1%)	4 (25.0%) 4 (25.0%) 1 (6.3%) 7 (43.8%)	0.45	3.50 (0.73–16.85) 2.04 (0.40–10.55.40.55) 1.17 (0.06–22.94) Reference
Fazekas Score (*N* = 131) Periventricular White Matter (median, IQR) Deep White Matter (median, IQR)	1 (0–2) 1 (0–1)	1 (0–2) 1 (0–2)	1 (0–2) 1 (0–1)	0.84 0.05	1.05 (0.74–1.50) **1.56 (1.01–2.41)**

IE indicates infective endocarditis; HIV Human immunodeficiency virus; HACEK Haemophilus, Aggregatibacter, Cardiobacterium, Eikenella and Kingella; CRP C-reactive protein; INR International normalized ratio. ^‡^Reference values are: 0.70–1.20 mg/dl for creatinine; <5.0 mg/l for CRP; 3.90–10.50/nl for leucocyte count; 150–370/nl for thrombocyte count; 0.90–1.25 for INR

Among IE-related parameters, patients with three-territory sign differed significantly regarding pathogens isolated from blood cultures and cardiac valves, as well as the valve affected by IE vegetations. A particularly strong association was observed between three-territory sign and the presence of extracerebral arterial embolic events. Elevated CRP on admission was likewise significantly associated with three-territory sign. In contrast, stroke-related factors and additional cMRI findings did not differ significantly between patients with and without three-territory sign

In patients with three-territory sign, Staphylococcus aureus was about twice as frequent (45.3% vs. 18.4%; odds ratio (OR) = 2.82; 95% confidence interval (95% CI) 1.03–7.71), the mitral valve was more frequently affected (62.8% vs. 44.9%; OR = 2.07; 95% CI 1.02–4.22) and extracerebral arterial embolic events occurred more often (66.2% vs. 30.8%; OR = 4.4; 95% CI 1.88–10.31). CRP values were nearly twice as high (median CRP on admission = 122.50 mg/l (IQR 45.60–232.60.60.60) versus 66.85 mg/l (IQR 35.28–147.63.28.63); OR = 1.01; 95% CI 1.00–1.01.00.01). In-hospital mortality was also significantly higher (31.4% vs. 12.2%, OR = 3.28; 95% CI 1.25–8.64). The association between three-territory sign and in-hospital mortality was robust in multivariable analysis (aOR = 3.14; 95% CI 1.17–8.43).

In multivariable logistic regression, three-territory sign was independently associated with Staphylococcus aureus (adjusted OR (aOR) = 3.98; 95%CI 1.35–11.75) and extracerebral arterial embolic events (aOR = 4.78; 95%CI 1.77–12.91) (Table 3). Multicollinearity was assessed for Staphylococcus aureus, mitral valve, extracerebral arterial embolism and CRP value on admission. Variance inflation factor was < 10 (around 1) for all four variables.

**Table 3:** Multivariable Analysis for the presence of three-territory sign

Parameter	Three-territory sign	No three-territory sign		*p*-value	Adjusted odd’s ratio	95% confidence interval
Age (median, IQR)	68 (55-?76)		67 (57–75)	0.90	1.00	0.97–1.04
Sex, male (n, %)	56 (65.1%)		36 (73.5%)	0.79	1.16	0.39–3.46
Staphylococcus aureus (blood culture)	39 (45.3%)		9 (18.4%)	0.01	**3.98**	**1.35–11.75**
Mitral valve affected	54 (62.8%)		22 (44.9%)	0.40	1.53	0.57–4.09
Extracerebral arterial embolism	43 (66.2%)		12 (30.8%)	0.002	**4.78**	**1.77–12.91**
CRP value (admission), mg/l (median, IQR)	122.50 (45.60–232.60.60.60)		66.85 (35.28–147.63.28.63)	0.26	1.00	1.00–1.01.00.01

CRP indicates C-reactive protein. Multivariable logistic regression analysis included patients with complete information on all variables in the model (*N* = 95). Analysis was performed with variables showing statistically significant differences in bivariate logistic regression analysis. In addition, it was adjusted for age and sex

## Discussion

Major findings of the study are: First, the three-territory sign on cMRI is common but not obligatory in IE patients with acute stroke. About one in three acute stroke patients with IE will not depict it. Second, presence of the three-territory sign is independently associated with a more virulent pathogen (Staphylococcus aureus) and additional extracranial embolization. Third, the three-territory sign indicates worse course of the disease connected to a significantly higher in-hospital mortality. Fourth, presence or distribution of other neuroimaging markers such as hemorrhagic stroke, CMB or chronic ischemic stroke are not strongly associated with the three-territory sign.

Our study provides useful data on frequency and significance of the three-territory sign in IE. Although common, about one in three acute stroke patients with IE will not depict three-territory sign if undergoing MRI. These patients appear to be less severely affected, as they less often have additional extracranial embolism and less often Staphylococcus aureus as underlying pathogen. Staphylococcus aureus is a pathogen common in IE and considered particularly virulent [2, 4, 5, 8, 22]. Identification of Staphylococcus aureus is also crucial to guide optimal therapeutic treatment. Presence of three-territory sign may encourage the clinician to even more thoroughly search for Staphylococcus aureus. Thus, infarct pattern may help decisions on diagnostic intensity.

Three-territory sign was also independently associated with extracerebral arterial embolism. This adds to a previous study, showing extracerebral embolic events to be more frequent in IE patients with stroke than without stroke [7]. Our findings corroborate studies that found a positive association between presence of acute stroke and further embolic events in IE [22, 23]. Our study adds to the literature, that within stroke patients with IE, in particular those with three-territory sign are at high risk.

Imaging markers such as accompanying hemorrhagic stroke, CMB or chronic infarcts were not more common in IE patients with three-territory sign, nor was a specific distribution of them. This suggests that hemorrhagic stroke, CMB and markers of chronic ischemia are rather separate entities as they do not concur with three-territory sign.

The significance of the three-territory sign as an indicator for severity of the disease is supported by higher CRP levels in patients with three-territory sign. This result substantiates previous findings where CRP values were higher in IE complicated by stroke, other embolic events and death [24, 25]. CRP indicates severity of the disease not only in IE in general but also in the subgroup of patients with IE and stroke.

Mortality, especially short-term mortality is higher in IE patients with stroke than in IE patients without stroke [2, 4, 23, 26]. Our study shows that in-hospital mortality is particularly high among IE stroke patients with three-territory sign. Thus, three-territory sign further reflects overall disease severity in this subgroup of patients. Assessment of stroke symptoms and severity, however, is hampered by the retrospective design of the study. Not all patients had an NIHSS score, and not all of the examiners had an NIHSS certificate. The association between three territory sign and mortality may therefore be mediated by higher clinical stroke severity in these patients.

Taken together, evaluation of stroke pattern may add distinct information for risk stratification in IE patients with concomitant stroke. Presence of three-territory sign on cMRI may be a marker of disease severity and, thus, may guide diagnostic approaches in clinical practice to identify patients at risk.

### Strengths and limitations

This study provides important new insights on the significance of the three-territory sign on cMRI in patients with acute stroke and IE. Work-up included a meticulous evaluation of acute and chronic infarcts, CMBs, ICH, subarachnoid hemorrhage and hemorrhagic transformation. The retrospective study design inherently has several limitations that must be considered. Selection bias (when performing an MRI or systematic extracerebral imaging) applies. Even though sex, age and in-hospital mortality did not differ significantly between IE patients undergoing cMRI or not, further factors that might have influenced acquisition of cMRI (e.g. hemodynamic stability, intensive care unit status, coma) have not been assessed. Clinically stable patients and patients with suspected cerebral embolic events may be overrepresented. Timing of MRI furthermore may have influenced the appearance of lesions on different sequences in our study. Additional limitations due to retropsective nature of the study are residual confounding, detection bias, and inconsistent or missing documentation. The limited sample size carries the possibility of a type II error. Based on our study, we therefore cannot establish causal relationships.

## Conclusion

A cerebral infarct pattern with ischemic lesions in all three vascular supply territories (three-territory sign) on cMRI is common but not obligatory in IE patients with acute stroke. In patients with stroke and IE, it depicts a high-risk subgroup with more frequent detection of Staphylococcus aureus, a particularly virulent pathogen, higher risk of additional extracerebral embolism and higher mortality.

## Data Availability

The datasets generated during and/or analyzed during the current study are available from the corresponding author on reasonable request. All authors had full access to all the data in the study and the corresponding author takes responsibility for its integrity and the data analysis.

## Abbreviations

DWI: Diffusion weighted imaging
ESC: European society of cardiology
GRE: Gradient echo
SWI: Susceptibility weighted imaging
EVT: Endovascular treatment
IVT: Intravenous thrombolysis
CRP: C-reactive protein
